# Effect of herbal extract granules combined with probiotic mixture on irritable bowel syndrome with diarrhea: study protocol for a randomized controlled trial

**DOI:** 10.1186/1745-6215-12-219

**Published:** 2011-10-06

**Authors:** Seok-Jae Ko, Bongha Ryu, Jinsung Kim, Beom-Gi Hong, Inkwon Yeo, Beom-Joon Lee, Jin-Moo Lee, Jae-Woo Park

**Affiliations:** 1 Department of Internal Medicine, College of Oriental Medicine, Kyung Hee University, Seoul, Republic of Korea; 2 Department of Statistics, College of Sookmyung Women's University, Seoul, Republic of Korea; 3 Department of Internal Medicine, Kangnam Korean Hospital, Kyung Hee University, Seoul, Republic of Korea; 4 Department of Oriental Gynecology, College of Oriental Medicine, Kyung Hee University, Seoul, Republic of Korea

## Abstract

**Background:**

Irritable bowel syndrome (IBS) is a chronic gastrointestinal disorder characterized by abdominal pain and change of bowel habits without organic disease. Many patients seek alternative IBS treatments because of the limitations of conventional treatments. *Gwakhyangjeonggisan *(GJS), a herbal formula, has long been used for alleviating diarrhea-predominant IBS (D-IBS) in traditional medicine. Duolac7S, which comprises 7 bacterial species as probiotics, has been frequently used for D-IBS. Although GJS and Duolac7S have been administered simultaneously in many D-IBS patients, no study has investigated the effects of GJS and Duolac7S combination therapy on D-IBS.

**Methods/Design:**

The current trial is a randomized, double-blinded, placebo-controlled, 4-arm study. After a 2-week run-in period, 60 patients with D-IBS will be randomly assigned to one of the 4 combination groups consisting of GJS (water extract granules, 3 g/pack, 3 times a day) with Duolac7S (powder form, 1 capsule, 2 times a day) or their placebos and followed up for 2 weeks. The assigned treatments will last for 8 weeks. The primary outcomes are adequate relief of IBS pain and discomfort and the proportion of responders (on a weekly basis). The secondary outcomes are visual analog scale for IBS symptoms (on a daily basis), quality of life (at 0, 8, and 10 weeks), intestinal permeability, and composition of intestinal microbiota (at 0 and 8 weeks).

**Discussion:**

The present study is designed to examine the safety and efficacy of GJS and Duolac7S combination therapy on D-IBS. Our study provides the clinical evidence of a new therapeutic strategy for D-IBS.

**Trial registration:**

ClinicalTrials.gov NCT01342718 .

## Background

Irritable bowel syndrome (IBS), a common chronic gastrointestinal disorder characterized by abdominal pain and alteration of bowel habits in the absence of structural abnormality, has a prevalence of approximately 15% in western populations [1,2]. Patients with IBS can be classified by their predominant bowel habits: diarrhea-predominant IBS (D-IBS), constipation-predominant IBS, or IBS with alternating bowel movements [3]. Although smooth-muscle relaxants, bulking agents, and anti-diarrheal agents are commonly used as conventional IBS treatments, many IBS patients turn to alternative treatments because of the lack of therapeutic advantages of these treatments [4]. Therefore, the development of a new therapy is necessary for IBS patients.

*Gwakhyangjeonggisan *(GJS; *Kkako-shoki-san *in Kampo Medicine; *Huoxiang-zhengqi-san *in Traditional Chinese Medicine), which was recorded originally in the famous ancient herbal formula literature *"Formularies of the Bureau of people's Welfare Pharmacies"*, consists of 13 common crude herbs. GJS contains the chemical ingredients naringin, hesperidin, thymol, honokiol, and magnolol [5] and has been shown to protect intestinal barrier function [6], contract the colonic muscle [7], and regulate infectious diarrhea [8]*in vivo*. In traditional Korean medicine, this herbal formula has long been used for relieving abdominal pain, diarrhea, and vomiting as an over-the-counter or prescribed medicine [9-11]. However, there have been no clinical trials to investigate the efficacy of GJS in IBS.

Probiotics are defined as viable microorganisms, which confer potential health benefits on the host when taken in proper amounts [12]. They are easily available, do not require a prescription, and are administered extensively for the relief of abdominal symptoms [13]. According to a recent systematic review and meta-analysis study, probiotics caused a modest improvement in the overall IBS symptoms [14]. The rationale for using probiotics for IBS is based on the assumption that they modify the composition of the intestinal microflora [15] and regulate intestinal permeability by modulating the epithelial tight junctions [16]. Duolac7S, a probiotic mixture, contains 7 bacterial species including *Bifidobacterium, Lactobacillus*, and *Streptococcus*. Each of these bacterial species is reportedly beneficial for IBS [17]. Duolac7S has been approved by the Korean Food and Drug Administration for restoring the ecological balance of intestinal microflora and improving intestinal symptoms [18]. Although Duolac7S has been widely used as an over-the-counter product for IBS-related symptoms, no clinical trials have investigated the efficacy of Duolac7S on IBS.

Recently, several herbal formulas and probiotics have been simultaneously administered to IBS patients for relieving abdominal symptoms [19]. In addition to lack of clinical evidence for the use of GJS or Duolac7S, the efficacy of their combined treatment for IBS has not been elucidated. Thus, there is a need to evaluate the safety and efficacy of administration of GJS, Duolac7S, or a combination therapy as a frequently used treatment for IBS. In the current trial, we will identify the safety and efficacy of GJS, Duolac7S, or a combination therapy on D-IBS by evaluating IBS symptoms and quality of life. To investigate their mechanisms in humans, intestinal permeability and composition of intestinal microbiota will be assessed.

## Materials and methods

### Objectives

The aims of the study are as follows: (1) to verify the effect of GJS combined with Duolac7S on D-IBS; and (2) to analyze mechanisms and establish scientific evidence for the use of GJS with Duolac7S by investigating changes in intestinal permeability and the composition of intestinal microbiota.

### Hypothesis

We hypothesize the following possibilities: (1) GJS combined with Duolac7S will show more beneficial effects on D-IBS symptoms than either GJS or Duolac7S alone; and (2) after 8 weeks of administration of GJS with Duolac7S, intestinal permeability will be lowered and the proportion of lactic acid bacteria in the intestine will be changed.

### Design

The current study will be conducted as a placebo-controlled and double-blind trial with 60 patients being randomly allocated to 4 arms. The trial will be implemented at Kyung Hee University Hospital at Gangdong in Seoul, Korea.

Participants will be required to pursue a 2-week run-in (weeks -2 to 0), 8-week administration (weeks 0 to 8), and a 2-week follow-up period (weeks 8 to 10) and to record daily symptoms in a diary.

**Figure 1:**
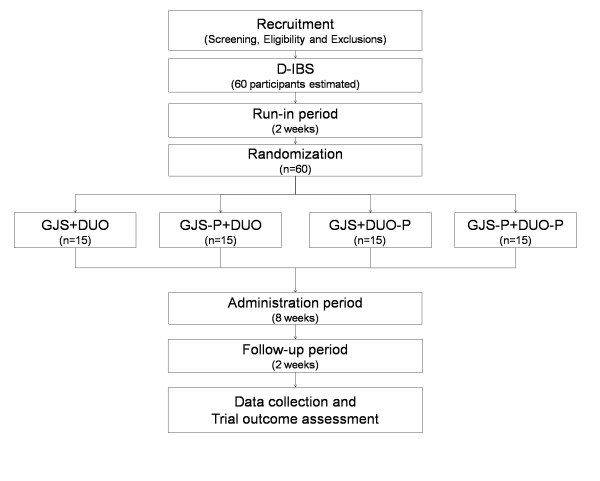
**Flow chart of trial**. D-IBS: Diarrhea-prediminant irritable bowel syndrome. GJS: *Gwakhyangjeonggisan*. DUO: Duolac7S. GJS-P: Placebo of *Gwakhyangjeonggisan*. DUO-P: Placebo of Duolac7S

During the administration period, 1 pack 3 times a day (2 h after each meal) of GJS or its placebo and 1 capsule 2 times a day (2 h after breakfast and dinner) of Duolac7S or its placebo will be provided for 8 weeks. All participants will be divided into 4 groups: (1) the real GJS and real Duolac7S group; (2) the real GJS and placebo Duolac7S group; (3) the placebo GJS and real Duolac7S group; and (4) the placebo GJS and placebo Duolac7S group. The flow of the entire trial is shown in Figure 1.

The current study will be performed in accordance with the standards of the International Committee on Harmonization on Good Clinical Practice and the revised version of the Declaration of Helsinki. The protocol of the trial has been approved by the institutional review boards (IRBs) and ethics committee of Kyung Hee University Hospital at Gangdong. The permission number of IRBs is KHNMC-OH-IRB 2010-011, and the protocol identification number of ClinicalTrials.gov is NCT01342718. Written informed consent will be obtained from all participants prior to enrollment and patients will be given adequate time to decide if they wish to participate before signing the consent form.

### Sample size calculation

The present trial has a pilot character of a new therapeutic strategy of GJS and Duolac7S. Similar to the results of a previous study, 12 participants who complete the study can be considered minimal clinical significance [20]. The drop-out ratio is assumed to be 20%. Ultimately, each group will consist of at least 15 participants so the total sample size should be more than 60 participants. This sample size is determined to provide 80% power to demonstrate the superiority of the study agents compared to placebos and a *P*-value < 0.05 will be considered statistically significant.

### Inclusion criteria

Patients aged 18-75 years with consistent abdominal pain and discomfort should meet the Rome III criteria of IBS [3]. Among IBS subtypes, participants must also be included in the D-IBS type, defined as loose (mushy) or watery stools ≥ 25% and hard or lumpy stools < 25% of bowel movements [3]. The Bristol scale, which is a visual descriptive stool form scale, will be used to assess stool consistency [21]. Patients should not have had structural abnormalities in their intestine detected by colonoscopy in the past 5 years.

### Exclusion criteria

Patients who report the following conditions will be excluded: (1) history of abdominal surgery which was related to IBS symptoms; (2) alarm symptoms such as severe weight loss in the past month, melena, and dysphagia; (3) gastrointestinal organic lesions such as cholangitis, pancreatitis, enteritis, and ulcer; and (4) severe systemic organ diseases such as cancer. All participants will have to undergo routine tests of complete blood count (CBC), kidney function (blood urea nitrogen and serum creatinine), liver function (serum aspartate transaminase and alanine transaminase), thyroid function (thyroid stimulating hormone), serum C-reactive protein (CRP), and stool occult blood in order to exclude those who have infection or serious organ diseases. Women with childbearing potential will be required to have a negative pregnancy urine test. Patients who have taken antibiotics, herbal medicine, or probiotics 2 weeks prior to screening will also be excluded.

### Recruitment

Information will be sent by mail to patients with IBS who were previously identified at the Kyung Hee University Hospital at Gangdong, and public advertisements will be placed in the newspaper to recruit participants. Colored fliers, brochures, and banners have been placed inside the hospital. We also plan to place advertisements on the homepage of the hospital and the boards of social networks like Twitter.

### Randomization

Randomization will be performed by an independent statistician by generating allocation numbers by using a random number creation program. The investigator will be notified of the number assigned to a participant via fax or e-mail.

### Blinding

The patients will be blinded to the treatment they are provided. The investigator, clinical research coordinator, and clinical pharmacist will also be blinded to randomization. Only the independent statistician will be associated with the randomization, and the entire procedure will be monitored by the authorized clinical research organization (CRO), Marinet Corporation, Seoul, Korea.

### Interventions

GJS, a famous herbal formula in Korea, has been traditionally used for managing various gastrointestinal dysfunctions including diarrhea, abdominal pain, and discomfort [9-11].

The GJS that will be used in this study is a brown, bitter herbal extract granule (Gwakjungsan granule^®^, Hanpoong Pharm & Food Co., Ltd., Jeonju, Korea) produced in accordance with the Korean Good Manufacturing Practice. Gwakjungsan granule^® ^(GJG), a water-extracted GJS mixed with starch and lactose, is allowed and regulated by the Korean Food & Drug Administration. Each GJG contains the 13 herbs listed in Table 1. All herbs will be obtained from qualified suppliers in Korea and the GJG will be sealed in opaque aluminum bags. Placebo GJG, made with cornstarch powder with the same color and taste as GJG, will be packed identically to the active herbal formula in opaque aluminum bags with the same labeling as real GJG. All packages will be dispensed by an independent pharmacist in a separate room. Patients will be requested to dissolve GJG or placebo granules in each package using water and take them 2 h after each meal. Voucher specimens of GJG will be retained at the research laboratory of the manufacturer.

**Table 1:** Ingredients of herbal formula (*Gwakhyangjeonggisan*)

Scientific name	Parts of use	Gram/day
*Agastache rugosa*	Above-ground parts	1.0
*Perilla frutescens*	Leaves and twigs	1.0
*Angelica dahurica*	Root	1.5
*Areca catechu*	Peel	1.0
*Poria cocos*	Dried core	3.0
*Magnolia officinalis*	Peel	2.0
*Atractylodes macrocephala*	Peel and rhizome	3.0
*Citrus unshiu*	Peel	2.0
*Pinellia ternate*	Stem	3.0
*Platycodon grandiflorum*	Root and peel	1.5
*Glycyrrhiza uralensis*	Rhizome	1.0
*Zingiber officinale*	Rhizome	1.0
*Ziziphus Jujuba*	Fruit	2.0

Duolac7S (Cell Biotech Co., Ltd., Gimpo, Korea) is a probiotic mixture containing multiple strains of 3 viable bacteria species: 3 strains of *Bifidobacterium *(*B. brevis, B. lactis and B. longum*); 3 strains of *Lactobacillus *(*L. acidophilus, L. plantarum, and L. rhamnosus*); and 1 strain of *Streptococcus *(*S. thermophilus*). Duolac7S will be contained in the capsule form with each capsule containing 5 billion bacteria in the powder form. Placebo Duolac7S, a powder consisting of cornstarch with the same color and taste as Duolac7S, will be contained in a capsule identical to the study agent. Patients will swallow 1 capsule of Duolac7S or placebo 2 h after breakfast and dinner for the 8-week administration period. At the end of the study, participants will be asked whether the herbal formula or probiotic that was administered was real or whether it was a placebo to evaluate the success of blinding.

Compliance will be checked by counting returned GJG or Duolac7S. A patient's data will be excluded from *per-protocol *analysis unless compliance reaches at least more than 60% of each GJG and Duolac7S.

All participants will be asked to record any adverse events in their diary during administration and the follow-up period. The CBC, CRP, renal function, and liver function will be assessed to determine the safety of treatments after the administration period. Analyses will be undertaken at an accredited laboratory. All values detected in the laboratory will be recorded by the investigator in the case report form.

### Rescue therapy and concomitant medications

During the entire clinical trial, patients will be prohibited from taking any kinds of drugs or therapies that might affect symptoms related to D-IBS. However, patients are allowed to take a specified medication for abdominal symptoms based on the doctor's judgment as a rescue drug if their D-IBS symptoms are exacerbated beyond a tolerable level. In such cases, the investigator will be informed of the rescue drug administration or it will be documented in their diary.

Patients must report all prescribed and over-the-counter medications taken during the study in their diaries. If patients receive antibiotics for acute infections, symptom data during the time of antibiotic administration and 1 week after completion will be excluded from analysis.

### Outcome measurements

#### Primary outcomes

Adequate relief (AR) will be used as a primary outcome to evaluate IBS symptom improvement. Patients are expected to report on a weekly basis their response to the following question: "In the past 7 days, have you had adequate relief of your IBS pain and discomfort?" AR will be measured at the end of each week during run-in, administration, and follow-up periods. Responders will be defined as patients reporting AR for at least 50% of the study weeks, which was applied as a major variable in previous studies [20,22]. The proportion of responders, another primary outcome, will be assessed individually during run-in, administration, and follow-up periods.

#### Secondary outcomes

Patients will be asked to complete a daily diary to evaluate the severity of the individual symptoms (abdominal pain, abdominal discomfort, bloating, flatulence, urgency, and mucus in the stool) and the severity of overall symptoms on a 100-mm visual analog scale (VAS) during run-in, administration, and follow-up periods [22]. The VAS scores are rounded to the nearest integer in millimeters. Patients will also be requested to fill in a daily diary to assess stool frequency and bowel functions on the basis of the consistency defined by the Bristol stool scale [21] and the ease of passage, as defined by the adjectival scale (from manual disimpaction to incontinence) [23].

A quality of life measurement for persons with irritable bowel syndrome (IBS-QoL) is a 34-item questionnaire assessing the degree to which IBS interferes with the patient's quality of life. The IBS-QoL is composed of 8 dimensions, including dysphoria, interference with activity, body image, health worry, food avoidance, social reaction, sex, and relationships. Each item is evaluated on a 5-point Likert scale with a higher score indicating a better quality of life [24]. The IBS-QoL will be filled out at the administration period baseline (week 0), the administration period end (week 8), and the end of the follow-up period (week 10).

#### Assessment of intestinal permeability

Intestinal permeability reflects the intactness of the intestinal mucosal barrier, which regulates the passive permeation of the luminal contents [25]. It is possible to assess intestinal permeability by evaluating the urinary excretion of lactulose and mannitol given orally [26]. Lactulose is only slightly absorbed and serves as a measure of the mucosal integrity while mannitol is easily absorbed and serves as a measure of the transcellular uptake [27]. The lactulose/mannitol (L/M) test is nontoxic and noninvasive in humans and is a stable measurement that is not affected by gastric emptying, intestinal motility, and renal function [28]. Therefore, the L/M test is a relevant method to measure intestinal permeability in the current trial. An increased ratio of the excretion of lactulose to mannitol suggests increased intestinal permeability [26]. Increased intestinal permeability is reported in various intestinal diseases associated with diarrhea such as leaky gut syndrome [25] or IBS [29,30].

The evaluation procedure is as follows: after an overnight fast, each subject will void to empty his or her bladder and then ingest 13.9 g of lactulose together with 5.3 g of mannitol dissolved in 500 mL of water. Urine will be collected over the next 8 h in plastic bags. Patients will drink approximately 2 L of water during the test, and other foods or liquid will not be allowed. The entire urine volume will be measured for 8 h and a 45-mL urine sample will be stored at -70°C until assayed. The urine will be collected at the baseline (week 0) and at the end of the administration (week 8) period.

#### Pattern analysis of intestinal microbiota

Associations have been observed between IBS patients' symptoms and the presence or quantities of certain gastrointestinal bacteria [31]. In the current study, we will focus on the change in the composition of the intestinal microbiota after administration of the study agents. Each subject will be expected to submit 5 g of fecal samples for bacteriological studies at the baseline (week 0) and at the end of the administration (week 8) periods. The samples will be collected immediately before urine collection and stored at -70°C prior to analysis. The fecal samples will be analyzed in a qualified laboratory (Cell Biotech Co., Ltd., Seoul, Korea) by the denaturing gradient gel electrophoresis (DGGE) method and real-time polymerase chain reaction (RT-PCR), which is a useful genotypic technique for detecting changes in the microbiota [32].

### Quality control

To maintain the accuracy and quality of the clinical trial, audits and monitoring will be implemented by the Marinet Corporation, a CRO located in Seoul, Korea. Clinical research associates will regularly monitor whether the clinical trial is proceeding based on the protocol by checking trial master files, informed consent forms, case report forms, adverse events, and compliance with study agents.

### Statistical analysis

Statistical analysis of all data will be performed by a specialized statistician in a blind manner. To assess the efficacy of this trial, both the *intention-to-treat *and *per-protocol *populations will be analyzed. Baseline characteristics will be compared by Pearson's chi-square test and analysis of variance (ANOVA). The target variables for analysis in this study include the following: (1) AR and proportion of responders (primary outcomes); (2) VAS for abdominal pain and diarrhea; (3) bowel function scores (frequency, consistency, and ease of passage); (4) severity of individual symptoms (abdominal pain, abdominal discomfort, bloating, flatulence, urgency, and mucus in the stool) and overall symptoms; and (5) IBS-Qol. All values will be presented as mean ± SEM. Comparison between the 4 groups will be performed on the basis of repeated measure ANOVA, and the paired *t*-test will be used to compare between the baseline and after a certain period in each group. The analysis for characteristics such as gender and age between groups will be conducted along with Pearson's chi-square test. All statistical analyses of the data will be performed using the SPSS program, version 16.0 (SPSS Inc., Chicago, IL) and a *P *value < 0.05 will be considered statistically significant.

## Discussion

Herbal medicine has been considered safe and effective for various diseases based on clinical experiences accumulated for thousands of years in traditional medicine and has gained attention as an alternative treatment for hard-to-cure or cryptogenic diseases such as IBS [33,34]. Despite recent enthusiasm for herbal medicine, there are only few well-designed randomized controlled trials related to herbal medicine, and its mechanisms have been poorly understood. In the current study, we will evaluate the efficacy of GJS, a representative herbal formula that has long been used for managing diarrhea and abdominal pain in traditional Korean medicine [9-11]. In addition, we will evaluate intestinal permeability and the composition of intestinal microbiota as biomarkers to assess the efficacy of GJS. The results of these markers are expected to make it possible to analyze the mechanisms and build up scientific evidences of GJS.

In addition to frequent usage as an herbal formula for IBS, probiotics are one of the promising agents extensively used for IBS. Probiotics have shown effects on IBS through a variety of mechanisms like competing with pathogens for nutrients and receptors, inducing hydrolysis of toxins and receptors, inducing production of organic acids, and modulating nitric oxide synthesis [35]. One notable mechanism of probiotics is the anti-inflammatory effect [36]. Approximately 15% of IBS patients may have a persistent mild inflammatory state resulting in increased intestinal mucosal permeability [37]. The following anti-inflammatory effects of single strains have been reported: *Lactobacillus plantarum *demonstrates significant benefits by increasing interleukin 10 synthesis and secretion in macrophages and T cells derived from the inflamed colon [38]; and *Bifidobacterium breve *and *Streptococcus thermophilus *release metabolites that exert an anti-tumor necrosis factor-alpha effect capable of crossing the intestinal barrier [39]. Duolac7S, a study agent in the current trial, contains these types of bacterial species and is expected to have an anti-inflammatory effect that will improve intestinal permeability.

Multi-strain probiotics and high-dose probiotics appear to have greater efficacy than single strains and relatively low-dose probiotics [40] and several probiotic mixtures can lead to the improvement of IBS symptoms. A probiotic mixture of 4 bacterial species (5 × 10^9 ^CFU per day) reduced abdominal pain and distension [41]. The probiotic VSL#3, which is a combination of 8 bacterial strains (4.5 × 10^9 ^CFU per day), has shown beneficial effects on flatulence and abdominal bloating [20,22]. Compared to previous study agents, Duolac7S contains a relatively high dose (1.0 × 10^10 ^CFU per day) of probiotics and various types of bacterial species (7 bacterial species). Thus, Duolac7S is anticipated to have a greater effect on IBS symptoms than other single strains or low-dose probiotics.

Predominant bowel dysfunctions, including diarrhea and constipation, can be associated with changes in the intestinal bacterial flora. One case-control study reported a lower concentration of bifidobacteria and a higher concentration of enterobacteriaceae in patients with IBS [42]. Another study using PCR-DGGE showed a higher concentration of coliforms in the IBS patients [43]. A study using high-throughput 16S ribosomal RNA sequencing demonstrated greatly reduced numbers of lactobacilli and collinsella in stool samples of IBS patients and an especially low concentration of bifidobacteria in D-IBS compared to controls [44]. Despite the limitations of these previous studies such as differing patient groups and the complexity of the intestinal microbiota, lactobacilli and bifidobacteria appear to be relatively reduced in patients with IBS. Thus, we assume that Duolac7S, which contains a large dose of lactobacilli and bifidobacteria, will modify the patterns of the intestinal microbiota and have a therapeutic effect on IBS symptoms and improve intestinal permeability by strengthening the integrity of the gut barrier.

The current study has several distinctive features. First, to the best of our knowledge, this will be the first trial to investigate the efficacy of GJS and Duolac7S combination therapy on D-IBS. In spite of the beneficial effects of GJS on IBS in traditional medicine, treatment of IBS by GJS has not been evaluated in clinical trials. No studies have targeted Duolac7S, a mixture of 7 lactic acid bacterial species, as a treatment for D-IBS; however, several studies have suggested that the individual bacterial species in Doulac7S and other probiotics mixtures may improve IBS symptoms. Moreover, GJS and Duolac7S combination therapy has never been the subject of a previous study. Thus, it is expected that the present trial will provide evidence for the effective application of GJS and Duolac7S combination therapy in D-IBS. Second, the participants in the current trial will be grouped into 4 arms. Test groups in this study will be divided into 4 arms in order to identify the efficacy of the combination therapy and to compare each treatment's effect. Ultimately, the combination therapy of GJS and Duolac7S are postulated to generate a synergistic effect resulting in greater improvement of D-IBS symptoms than individual GJS or Duolac7S treatment. Third, we will conduct analyses of intestinal permeability and the composition of intestinal microbiota after administration of GJS with Duolac7S. The urine and stool analysis results could provide evidence for the combination treatment and the possible mechanisms might also be investigated.

Although our study has a relatively small sample size for a pilot study, our trial is considered to have creative and challenging characteristics and is expected to play an important role in providing basic clinical data in the field of herbs and probiotics and developing methodology in IBS studies.

### Trial status

The study was conceived and designed in 2010. The first participant was randomized on 25 April 2011. The recruitment of the study is ongoing.

## Abbreviations

ANOVA: analysis of variance; AR: adequate relief; CBC: complete blood count; CRO: clinical research organization; CRP: C-reactive protein; DGGE: denaturing gradient gel electrophoresis; D-IBS: diarrhea-predominant irritable bowel syndrome; DUO: Duolac7S; DUO-P: placebo of Duolac7S; GJG: Gwakjungsan granule^®^; GJS: *Gwakhyangjeonggisan*; GJS-P: placebo of *Gwakhyangjeonggisan*; IBS: irritable bowel syndrome; IBS-QoL: a quality of life measurement for persons with irritable bowel syndrome; IRBs: institutional review boards; RT-PCR: real-time polymerase chain reaction; VAS: visual analog scale.

## Competing interests

The authors declare that they have no competing interests.

## Authors' contributions

SJK, JWP, IKY and BJL contributed to the funding for the study and to the design of the study. JSK, BHR, BGH and JML participated in the study design. SJK and JWP drafted the study protocol and prepared the manuscript. SJK, JWP and IKY were responsible for the statistical design of the study. All authors read and approved the manuscript.
